# Evaluation of Different Advanced Approaches to Simulation of Dynamic In Vitro Digestion of Polyphenols from Different Food Matrices—A Systematic Review

**DOI:** 10.3390/antiox12010101

**Published:** 2022-12-31

**Authors:** Carmen Duque-Soto, Alejandra Quintriqueo-Cid, Ascensión Rueda-Robles, Paz Robert, Isabel Borrás-Linares, Jesús Lozano-Sánchez

**Affiliations:** 1 Department of Food Science and Nutrition, Faculty of Farmacy, University of Granada, 18071 Granada, Spain; 2 Departamento de Ciencia de los Alimentos y Tecnología Química, Facultad de Ciencias Químicas y Farmacéuticas, Universidad de Chile, Santiago 8380492, Chile; 3 Department of Analytical Chemistry, Faculty of Science, University of Granada, 18071 Granada, Spain

**Keywords:** dynamic in vitro digestion, phenolic compounds, SHIME, TIM, SIMGI

## Abstract

Phenolic compounds have become interesting bioactive antioxidant compounds with implications for obesity, cancer and inflammatory gastrointestinal pathologies. As the influence of digestion and gut microbiota on antioxidant behavior is yet to be completely elucidated, and due to limitations associated to in vivo studies, dynamic in vitro gastrointestinal models have been promoted. A systematic review was conducted of different databases (PubMed, Web of Science and Scopus) following PRISMA guidelines to assess different dynamic digestion models and assay protocols used for phenolic compound research regarding bioaccesibility and interaction with colonic microbiota. Of 284 records identified, those including dynamic multicompartmental digestion models for the study of phenolic compound bioaccesibility, bioactivity and the effects of microbiota were included, with 57 studies meeting the inclusion criteria. Different conditions and experimental configurations as well as administered doses, sample treatments and microbiological assays of dynamic digestion studies on polyphenols were recorded and compared to establish their relevance for the dynamic in vitro digestion of phenolic compounds. While similarities were observed in certain experimental areas, a high variability was found in others, such as administered doses. A description of considerations on the study of the digestion of phenolic compounds is proposed to enhance comparability in research.

## 1. Introduction

Plants have been traditionally consumed for more than their nutritional value, as they have always been related with therapeutic effects [1,2,3]. This has been related to their bioactive properties, such as an outstanding antioxidant activity. The growing interest of the food and pharmaceutical industries in the development of more natural alternatives has stimulated the research into compounds promoting these health benefits. For this purpose, plant-derived bioactive compounds have been identified and isolated from different matrices [4,5,6,7]. These antioxidant compounds include plant secondary metabolites, which have been thoroughly evaluated for their potential activities in both in vitro and in vivo studies [8].

Among bioactive phytochemicals, polyphenols have risen as one of the most widely researched in the scientific community [9]. They have proven interesting bioactive and technological activities, as they pose as antioxidant, antimicrobial and antiviral molecules, which has been related to their structural features (mainly, the number and arrangement of the hydroxyl groups) [10,11,12,13,14,15,16,17,18,19,20,21,22,23]. Their consumption has also been linked to anti-inflammatory and anti-proliferative activities [24]. Additionally, a special interest has been taken in their impact on the modulation of the colonic microbiota profile, the role of gut microbiota in the bioaccesibility of phenolic compounds with its implications on their later antioxidant and bioactive properties in the colon and its close relation to health [25].

In order to exert their beneficial effects, these compounds must survive under gastrointestinal conditions. However, their labile nature and sensitivity to both low and high temperature pose as a challenge for their use [26,27]. Not only do technological processes present harmful conditions to these compounds but also the nature of their oral administration forces them to be submitted to gastrointestinal conditions (i.e., an acidic environment). This favors their degradation, hindering the consecution of their bioactivity. The metabolization of these compounds by the gut microbiota also favors this effect, altering the phenolic profile and therefore the observed antioxidant capacity [28]. From this perspective, it is essential to gain insight into the influence of different conditions on some aspects: their stability along the digestive tract and metabolization and their impact on bioaccessibility and bioavailability. These parameters are essential for understanding and improving their antioxidant activity once consumed.

In this way, gastrointestinal evaluation studies of these compounds focused on the impact of digestive conditions and the effects of the colonic microbiota are needed. Thus, it is necessary to represent as closely as possible the conditions of the digestive tract and to include an accurate representation of the gut microbiota to evaluate the evolution of their phenolic profile as related to their observed antioxidant activity and health benefits. Although in vivo studies have been proposed, their complexity related to technical difficulties, ethical problems and different parameters that may impact the observed results, such as gender, age or previous disorders, has motivated the search for alternatives [23,29]. Thus, in vitro gastrointestinal simulation models have risen to overcome those drawbacks, while searching for a closer representation of the in vivo situation [30,31].

Static in vitro models have been widely used among the scientific community to study the bioaccesibility and bioactivity of many compounds [32,33,34,35]. Even though these methods can provide great information on the digestion of simple foods and isolated or purified food components, they are considered as preliminary trials, as they are less applicable in comprehensive studies of the complete digestion process including colonic fermentations and lack a close representation of its dynamic nature [23], thus altering their digestion and not representing accurately the digestive process, which in turn deprives us relevant information of the phenolic profile and therefore antioxidant activity. Absorption, transport kinetics, the lack of representation of the mucosal barrier and peristaltic movements or continuous control and changes in pH and secretion flow rates are among the factors that are still to be represented in static models. Additionally, conditions for the specific digestion of phenolic compounds are not standardized. Therefore, a balance between simplification and accuracy in reproducing the physiological conditions must be considered [36].

Dynamic digestion models can represent the complex modulations of in vivo conditions and the different steps of the gastrointestinal process. The inclusion of the representation of colonic fermentation under dynamic conditions opens a gate to research on the chronic administration of bioactive compounds not only focused on their stability but also on the effect of microbial metabolization and their modulating effect over longer periods of time under representative conditions.

The aim of this work is to review different in vitro dynamic digestion models and assay protocols in the context of phenolic compound research as focused on the study of their bioactivity through their bioaccessibility along the digestive process and the interaction and modulation on colonic microbiota. Therefore, the different assay conditions, configurations, administered doses, sample treatments and microbiological assays applied in these studies have been evaluated for their relevance in the in vitro dynamic gastrointestinal digestion of phenolic compounds. To our knowledge, this is the first article focused on the thorough comparative review of the experimental conditions and related methodology of different dynamic multicompartmental models on the digestion of phenolic compounds.

## 2. Materials and Methods

This systematic review was conducted according to the Preferred Reporting Items for Systematic Reviews and Meta-Analyses (PRISMA) guidelines 2020 [37]. Comprehensive research of the electronic databases Pubmed, Scopus and Web of Science databases was performed by the authors for the selection of papers published until September 2022. Searches were made using combinations of the term “polyphenols” with “in vitro” and “dynamic” and “digestion” or “SHIME” or “TWINSHIME” or “TIM” or “simgi” or “AINIA” and “phenolic compounds” with “in vitro” and “dynamic” and “digestion”. These terms were selected in order to search for overall studies focused on dynamic digestion of the compounds of interest while also including specific research on articles regarding widely used and validated dynamic in vitro digestion multicompartmental models. Moreover, a manual search of articles referenced in selected papers was developed, considering the same eligibility criteria as further described in this section. As this review was focused on the experimental conditions and assays for different models, papers were grouped depending on the dynamic gastrointestinal model used, and duplicate articles were removed.

**Eligibility criteria**. These criteria were chosen to limit the considered articles to those focused on the subject of interest: the use of human-based dynamic in vitro multicompartmental models focused on the evaluation of different phenolic-rich sources during digestion, including colonic evaluations. Thus, selected articles were limited to those using dynamic in vitro multicompartmental models for the study of phenolic compound bioaccessibility or interaction with microbiota. Review articles, static digestion articles, studies using noncompartmental models and those using non-human models were excluded from the analysis. No language filter was applied.

## 3. Results and Discussion

### 3.1. Study Selection

**Figure 1:**
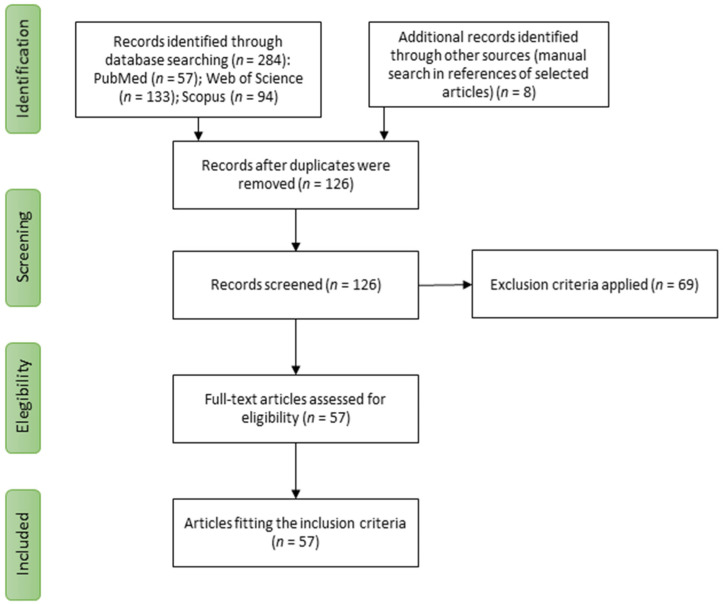
Study selection process for the systematic review.

The study selection process is shown in Figure 1. Based on the search strategy, 284 articles were found, of which 158 were duplicates and were excluded. Finally, 57 studies were eligible for inclusion in the systematic review with no additional articles identified from reference lists.

### 3.2. Dynamic In Vitro Digestion Models

As mentioned, in order to evaluate the realistic antioxidant and bioactive effect of phenolic compounds once consumed, it is vital to develop studies and conditions that are able to represent gastrointestinal conditions and the colonic microbiota community, as those are important factors in the observed results.

Due to the limitations of in vivo studies, which may situate them as less than ideal models, both static and dynamic in vitro digestion processes have been proposed. In particular, dynamic gastrointestinal models suppose a refinement in the development of ex vivo methods allowing for a closer representation of the characteristic dynamism of physiological conditions, with particular emphasis on gut microbiota. Indeed, as described above, the recent interest in the relation between phenolic compounds and colonic microbiota focused on their metabolization and modulation of the microbial profile has put forward the need for adequate models, which may allow for a close representation of the microbial composition to in vivo conditions [38].

Different approaches have been considered, including essential factors of the gastrointestinal tract: physical forces, geometrical considerations, chemical and enzymatic digestion. Different designs can be achieved through mimicking their spatial distribution and form, as it is related to the accurate simulation of physical forces (such as peristaltic movements) that could be of interest for gastric simulation. Additionally, biochemical digestion is achieved by developing simulated secretions and their addition through different digestive stages, either continuously or in specific moments throughout the process [39]. This constitutes an improvement from static models, where only one set of initial conditions is considered and changes in secretions over time are not contemplated.

An initial approach in dynamic digestion through analyzing and simulating individual compartments is the so-called noncompartmental model. This term includes gastric models, such as the Dynamic Gastric Model (DGM^®^), the Human Gastric Simulator (HGS^®^), the Gastric Digestion Simulator (GDS^®^) and the In Vitro Mechanical Gastric System (IMGS^®^) and colon models such as the Artificial Colon (ARCOL^®^) [39,40,41,42,43]. However, these may present some limitations. Firstly, although insight into specific processes is gained, it must be considered that the isolation of a specific digestion step may reduce the information we acquire to study the complete digestion process. By doing this, part of that dynamism is lost through the consideration of different steps as separate from the previous digestive process, such as the movement of the food bolus throughout different digestion chambers. However, noncompartmental models have been used for studying polyphenolic stability and bioaccessibility [44,45].

Consequently, the development of dynamic models considering more digestion steps was proposed. Multicompartmental models can range from the representation of the stomach and small intestine (DIGDI^®^; the TNO Gastro-Intestinal Model, TIM-1^®^; the Engineered Stomach and Small Intestine, ESIN^®^) to the inclusion of the colonic stages of digestion (the Simulator of Human Intestinal Microbial Ecosystem, SHIME^®^, the TNO In vitro model of the colon, TIM-2^®^; the Simulator of the Gastro-Intestinal tract, SIMGI^®^; the dynamic-colonic gastrointestinal digester (DGID-CF^®^)) [46,47,48,49,50,51]. These models are under a continuous process of optimization. Indeed, recent developments have included the consideration of an in-line oral step (as presented in ESIN^®^), the development of the simulation of infant or elderly gastrointestinal conditions or the inclusion of a mucosal phase, allowing then for the representation of the colonic microbiota associated with this structure.

Multicompartmental models have been of great interest in the study of the bioaccessibility of phenolic compounds. They allow for the study of stability, the impact of gastrointestinal conditions and the metabolization and bioaccessibility of these compounds throughout the digestive process as crucial factors related to their absorption. Moreover, the inclusion of a colonic stage allows for the additional consideration of the microbial metabolization of these compounds, which allows the consideration of this aspect and its possible correlation to their observed bioactivity, such as antioxidant activity [52,53]. This is possible thanks to this colonic stage in multicompartmental models, such as SHIME^®^, being validated for its accurate representation of the colonic microbiota [54].

Therefore, these models pose an incredible opportunity for the study of phenolic compound digestion. However, there are still differences in these representations that may affect the simulation, and consequently determine difficulties in the comparison of results. Thus, the evaluation of the conditions for the closer representation of the gastrointestinal process for phenolic digestion and microbial metabolization is needed in order to study their antioxidant effect once administered. Specific details involved in the dynamic simulation of the gastrointestinal process applied for the study of phenolic compounds will be discussed below.

### 3.3. Assay Conditions: Experimental Design

As described above, a variety of models for in vitro dynamic digestion have been developed. In this section, a comparative analysis between different studies applied for the in vitro dynamic digestion of polyphenols is discussed.

#### 3.3.1. Equipment Configuration

An abundance of dynamic gastrointestinal models has been used for the study of phenolic compounds, with differences in their representation of the digestive process that may influence the observed results. Thus, the selection of the used model and equipment configuration must be carefully accomplished to adapt conditions to the desired observations.

Firstly, we must distinguish between short-term and long-term intended models. One of the main models focused on short-term representations is the TIM^®^ system. The mentioned model possesses two separate units to represent the whole digestive process. On the one hand, TIM-1^®^ includes the simulation of the upper gastrointestinal tract presenting four different reactors for the stomach, duodenum, jejunum and ileum [55,56,57], whereas other systems only include the upper gastrointestinal tract [58,59,60,61,62]. On the other hand, TIM-2^®^ includes the simulation of the colonic area and its microbiota through the inclusion of four interconnected glass compartments, which simulate the first part of the colon (the ascending colon) continuously [63]. This separation of the digestive process into two distinct units allows for a focus on upper or lower gastrointestinal digestion. Furthermore, the use of the TIM-2^®^ model may be useful when upper gastrointestinal digestion does not significantly affect the administered sample, thus reducing the digestion to the colonic stage.

Other models are more focused on the development of long-term studies. Most of them represent three distinct parts of the colon. In this sense, they consider the 5-reactor system proposed by Molly et al., 1993, based on the Reading model described by Gibson et al., 1988, in which the representative three colon stages were validated [64,65]. Thus, different reactors are present for the stomach, small intestine, ascending colon (AC), transverse colon (TC) and descendent colon (DC), each with specific and constantly controlled conditions for the accurate representation of each area. This is the case for the SHIME^®^, SIMGI^®^ and DGID-CF^®^ models, as well as other dynamic gastrointestinal systems [66,67].

Moreover, the presented configuration can be modified. For example, the SHIME^®^ model allows for the inclusion of 10 reactors per unit (TWIN-SHIME^®^) [68,69,70,71,72,73,74,75]. Thus, two individual gastrointestinal lines may be included in the same equipment. Nevertheless, experiments using only five reactors have also been developed [73,74,76,77,78,79,80]. This flexibility may also admit the development of multiple other configurations depending on the interest of the study. In some studies, a reduction of colonic stages represented per line has been observed to introduce three different lines, known as TRIPLE-SHIME [52,81,82]. In that respect, the stomach and small intestine are still included as two separate processes in time, with different conditions for each one, but colon representation may be reduced to the Proximal Colon (PC) and Distal Colon (DC) [53]. In addition, further reductions in the represented reactors could be possible, including a stomach, small intestine and colon reactor per line. This reduced configuration may allow for the study of additional conditions simultaneously and prove to be a useful tool for screening purposes. However, due to this reduction the obtained results may not be as informative; however, they represent a great start for experimental design. Moreover, a representation of the mucosal layer is possible in this model, which may allow for the study of mucose-associated microbiota.

This flexibility is also present in the SIMGI^®^ model, where using separate compartments or sequential experiments is possible. As for polyphenols, some studies have eliminated the stomach representation from the experiment, beginning the intestinal representation directly on the small intestine. This is useful when previous evidence states the absence of any influence of stomach digestion over the studied compounds, thus focusing more on colonic microbial metabolism [83,84].

**Figure 2:**
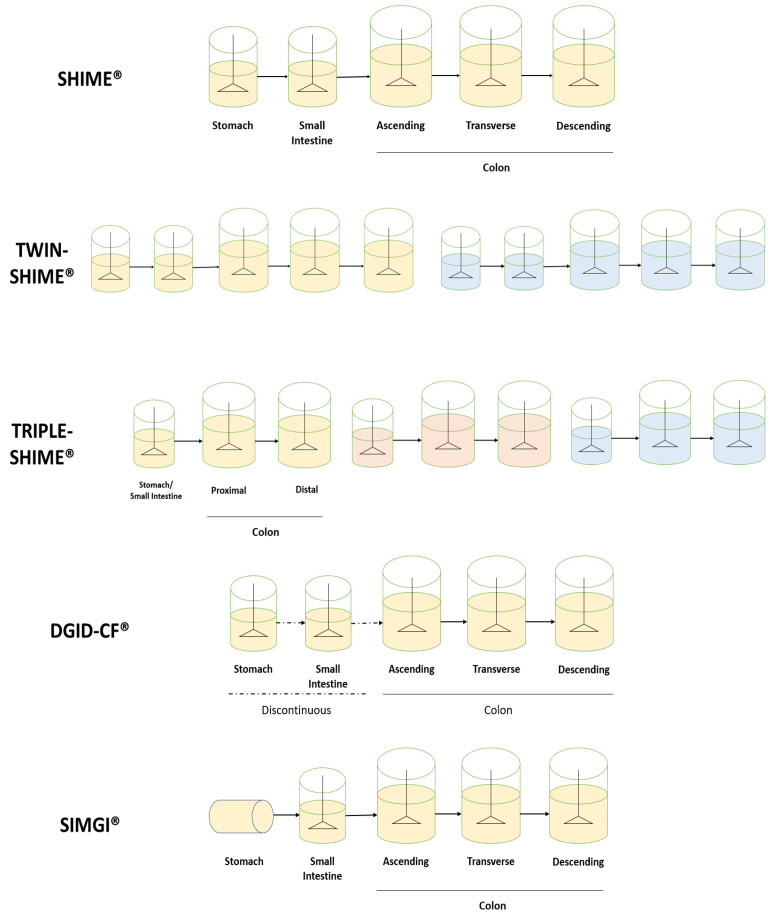
Experimental configurations of the described models.

The DGID-CF^®^ model also considers five vessels for the representation of the stomach, small intestine, ascending, transverse and descending colon [85]. Unlike the above-described, the system operates semi-continuously in the stomach and small intestine and continuously in the three colon regions. This structure may allow for the independent operation of the upper and lower gastrointestinal tracts. Configurations of the described systems are depicted in Figure 2.

Furthermore, the mentioned dynamic models have also been used in combination with static digestion. Thus, static upper gastrointestinal digestion or a pre-digestion of the studied compounds can be carried out for their later dynamic colonic digestion [86,87,88]. Alternatively, stomach and small intestinal digestion can be carried out dynamically, generating an intestinal digested extract (IDE) for their later static colonic digestion, which may use the equipment for this later purpose [89,90,91,92]. In addition, dynamic models have also been used as a source of representative microbiota to use as inoculum for static batch studies [64,65].

Additionally, the use of some of these models could allow for the simulation of specific digestive settings, including different species (human, dog, pig, calf), ages (infant, adult, elderly), pathologies and meal-related parameters.

Thus, the plasticity of these models shows the versatility and potential of the dynamic digestion models in the development of experimental configurations adapted to the needs of the study.

#### 3.3.2. Experimental Steps

As previously stated, dynamic digestion may include short-term or long-term studies, in which the overall duration of the experiment is variable. Generally, for the design of one of these experiments, it must be taken into consideration that a longer duration allows for a stronger emphasis on the ecological aspects on the colon microbiota.

Dynamic gastrointestinal digestion experiments, including colonic representation, present a series of distinct phases, which have been recorded in the reviewed articles. Generally, those phases involve a stabilization period in which the microbiota inoculum adapts to the model, a control period in which the microbiota is finally stable, a treatment period and finally a wash-out period [71,75]. To accurately observe the effects of the treatment, basal conditions must also be recorded through the obtention of samples during a time in which the microbiota is stable; that is the control period [68,69,71,73,74,75,77,78,79]. The wash-out period allows for the dilution of the effect of the treatment on the microbiota, while also giving us information on the duration of the observed effect when a dose is no longer administered [52,71,72,73,75,76,81,83,84,92,93]. Independently of their short or long-term nature, the mentioned phases may differ in duration or not be included at all.

In fact, an abundance of studies has used the SHIME^®^ model for the study of polyphenols. In these experiments, this structure is normally followed. As they mainly include long-term experiments, the adequate stabilization of colonic microbiota is essential. In this sense, the duration of this step is generally 2 weeks [70,74,75,76,77,78,79,80,81,82]. Nevertheless, as stabilization may differ depending on factors such as the microbiota inoculum, shorter and longer periods have also been recorded: from 1 week to 3.5 weeks [53,68,71,72]. Thus, the duration of this step is not rigid and may be extended until a stabilization of metabolism parameters from the microbiota is observed.

Then, a control period is usually carried out. Its duration ranges from one [68,71,77,78] to two weeks [69,74,75,79,82,94]. Stable microbiota conditions must be observed throughout this step and baseline samples are taken for later analysis. However, as a reduction in the duration of the experiment, some studies have not considered this stage or taken measurements through the previous days to the treatment phase [52,73,76]. Moreover, the introduction of multiple SHIME units introduces the possibility of the addition of an untreated simulated digestion as a control line submitted to the same conditions as the treatment line, except that the studied extract is not added. This may allow for the acquisition of baseline samples throughout the whole experiment [52,75,81].

**Figure 3:**
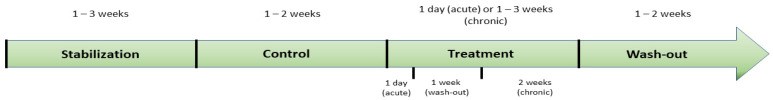
Timetable of acute and chronic experiment.

The duration of the treatment step is highly variable and may depend on different factors, such as long-term or short-term studies. It is also important to stress two different main approaches in the design of the treatment step, which may differentiate two types of studies, independently of the used model: acute [87] or chronic studies [52,69,72,73,74,75,76,77,78,79,81,82]. The general timetable of acute and chronic studies has been depicted in Figure 3. The main difference between these approaches is the frequency of administration of the sample under study. This factor also affects the duration of this experimental step, as acute studies only include one day of treatment, while the duration of chronic studies is commonly 2 weeks, but it can range from a few days [70] to 3 weeks [53,69,75]. A combination of both approaches could also be possible, with an initial single dose, an intermediate wash-out period of 1 week and a 2-week chronic dose period [68,71].

The duration and inclusion of the treatment step are highly dependent on the experimental configuration and the consideration of multiple treatment conditions. If a single experimental line is used and multiple treatments are considered, additional treatments could be included after sequential wash-out periods [79]. However, the model’s flexibility allows for multiple simultaneous experimental lines; thus, different samples may be included in different lines, reducing the overall duration of the whole experiment in the system. These configurations may also be interesting for the study of microbiota responses from different conditions or individuals under the same treatment [69].

However, in the study development in which a specific food matrix is considered as a mere vehicle for the administration of phenolic compounds, the microbiota could be adapted first to those conditions in order to ensure that the observed changes are not related to the higher presence of microbial substrates [77,78]. In this sense, a previous adaptation period using only the intended matrix should be added with generally a duration of 2 weeks, until the microbiota is fully adapted to these conditions. Additionally, the washout period also ranges between 1 and 2 weeks [71,72,73,75,76], although this period may be further reduced [52,53,81].

In SIMGI^®^, the described experimental steps have also been followed as stated above. In this sense, the stabilization period is also 2 weeks, but longer periods have also been observed, such as 19 days [67]. In these experiments, a control phase has not been stated but control experiments have been carried out, allowing for baseline observation throughout the whole process [95]. As previously stated for SHIME^®^, acute [83,84] and chronic [95] experiments can be developed with a duration of 1 day for the former and 2.5 weeks for the latter. A combination of both approaches can also be possible, which has been observed with a 2-week duration for chronic intake [84]. The average washout period in the reviewed articles is 1 week. The use of multiple administration periods with a similar approach as stated above may be used including multiple treatments after subsequent washout periods using multiple SIMGI^®^ units [83,84].

On the other hand, the DGID-CF^®^ model presents an experimental procedure consisting of two phases: stabilization and intake period. In phenolic compound studies using this model, the microbiota adaptation was similar to SHIME^®^ and SIMGI^®^, lasting up to 11–12 days. The intake period presented more variability, but it was in line with the other mentioned models, from 1–2 weeks [85,96].

Moreover, the possibility of the development of batch experiments in these models also needs to be considered, as seen for the SHIME^®^ and SIMGI^®^ models [70,90,91]. Additionally, stabilization and control periods may be excluded or shortened, focusing on the development of a treatment period with an overall duration of 48–72 h [70,89,91].

As for TIM-2^®^, timeframes are usually reduced in comparison to long-term experiments. The overall experimental duration could be up to 3 weeks, which has been the longest the system has been tested [97]. This is related to the presence of a dialysate system that prevents the accumulation of microbial metabolites. Nevertheless, experiments are usually performed over a 1-week period and a significant reduction in duration for all phases is observed; thus, short-term experiments can be developed. The adaptation of the microbiota to the system is normally achieved over 16 h. This has been extended in some cases to 20 h, but in all cases this period still remains under 24 h [88,98,99]. As already mentioned, the fact that long-term studies include a longer adaptation of the microbiota to the simulated conditions is related to a stronger emphasis on the ecological aspects of the digestion. These experiments do not usually present a control stage, but some may include simultaneous control experiments [100,101]. After adaptation, a 2–4 h starvation period is included, to allow for the fermentation of all the available carbohydrates in the system [98]. Then, a 72 h treatment phase is included, although shorter experiments have also been considered with 12–24 h [93,100].

Overall, while some consistency has been seen on the experimental steps present in studies using different models, variability is observed regarding their duration. In this area, an evaluation on the effective time to observe an effect resulting from the specific treatment could be interesting to standardize treatment times.

#### 3.3.3. Digestive Conditions

In this sense, many factors must be considered when deciding on a specific dynamic gastrointestinal system, such as the applied digestive conditions, which are different between models, or the control of specific parameters, which should also be considered throughout the process. For this purpose, the need for more clarity in some of the described methods and studies should be considered.

For SHIME^®^ experiments, most studies consider the experimental conditions described in Possemiers, 2004, including the reactor set-up, volume, residence time and pH conditions, as well as the nutritional medium composition [102]. In this sense, the most important control parameters are feed composition, pH and retention time, with an emphasis on pH and volume control in the colon vessels. In these experiments, the volume in the stomach vessel was 140–200 mL and for the small intestine 200 mL, which were of the fill-and-draw principle. A defined amount of SHIME^®^ nutritional medium (3 × 140 mL/day) and pancreatic and bile liquid (3 × 60 mL/day) was added to the stomach and small intestine vessels, respectively, with a residence time in each vessel of 2–4 h for the stomach and 4 h for the small intestine [68,69,71,72,73,74,75,76,77,79,103].

In the first two reactors, a specific control of pH was not established, but pH 2 was set in the feed [73,102]. However, some studies have included a pH range for stomach and small intestinal digestion, such as 2.0–2.5 and 5.0–6.0, respectively, as seen in Chen et al., 2022, or 5.0–5.5 for the intestinal vessel in Attri et al., 2018 [73,76]. As can be observed, this range may be variable depending on the study. Feed was directly introduced in the stomach compartment, while artificial pancreatic and bile liquids were introduced in the small intestine reactor.

The AC, TC and DC vessels were continuously stirred and presented a total volume of 500, 800 and 600 mL, respectively. Nonetheless, a reduction to half of the stated volumes can also be applied [70]. Their overall residence time was 76 h, consisting in 20 h AC, 32 h TC and 24 h DC, although the distribution per colon reactor may vary [76]. In addition, each of the colon compartments presents an established pH range, which is controlled throughout the experiment: 5.6–5.9 for AC, 6.1–6.4 for TC, 6.6–6.9 for DC. Anaerobic conditions were maintained by flushing with N_2_, and temperature was kept at 37 °C [58,59,71,72,73,74,75,76,77,103].

However, when proximal and distal colon reactors were considered, volumes were 500 mL and 800 mL, respectively. In this scenario, pH was adjusted as 5.6–5.9 for the proximal colon and 6.6–6.9 for the distal colon [52,53,81,82].

In most of the SHIME^®^ experiments, the inoculum was prepared as described in De Boever et al., 2000 [104]. For fecal slurry preparation, 10 g of fecal sample was diluted and homogenized with 100 mL sterilized phosphate buffer (0.1 M, pH 7) containing 1 g/L sodium thioglycolate as reducing agent. This was centrifugated (1 min, 500× *g*) and, in the case of studies in which more than one donor was used, supernatants were pooled [76,79]. In the above-mentioned study and other studies [68,69,71,74,77,78,79,81,104], 50 mL was introduced into the last three vessels, although inoculated volumes may vary (5–10% of the total content in each reactor or 40 mL) [73,82,103].

For SHIME^®^, the nutritional medium may vary depending on the experiment, but the composition seems to be similar, as presented in Table 1.

As for TIM-2^®^, in its four interconnected glass reactors, the thermostatic water located between the glass jacked and the membrane is kept at body temperature (37 °C for humans). As this system only simulates the conditions occurring in the proximal colon, pH is kept at 5.8 or above by the secretion of 1M NaOH when necessary. Additionally, it is possible to represent the complete colon by programming a pH gradient over time from 5.8 in the AC, 6.4 in the TC and 7 in the DC. In this sense, volume and pH are thoroughly controlled throughout the process. Anaerobic conditions are maintained by flushing N_2_. In experimental papers, the total volume of the system is not clearly stated.

As for the preparation of the fecal slurry, the protocol described in Venema et al., 2000, is usually followed for the use of pooled microbiota [105]. For this purpose, fecal samples (500 g) are mixed with concentrated dialysis liquid (per liter: 2.5 g K_2_HPO_4_⋅3H_2_O, 4.5 g NaCl, 0.005 g FeSO_4_⋅7H_2_O, 0.5 g MgSO_4_⋅H_2_O, 0.45 g CaCl_2_⋅2H_2_O, 0.05 g ox-bile, 0.4 g cysteine hydrochloride; pH 5.8), demi-water and glycerol as a cryoprotective agent. This mixture is homogenized under strictly anaerobic conditions. As for inoculated volume, this seems to be variable, ranging from 30 mL [106] to 70 mL [88,98,99] for all phenolic studies.

As for the nature of the nutritional medium, this model is fed with a Simulated Ileal Efflux Medium (SIEM), which mimics the composition present in the terminal ileum, depicted in Table 2. Modifications to this composition can be made depending on the specific study. For the study of polyphenols, these compounds are added to the standard medium and this mix is introduced into the TIM-2^®^ system during the treatment period [65,105,107,108].

In SIMGI^®^, initial volumes for the stomach and small intestinal compartments have been recorded as 65 and 55 mL [89,92], with a later addition of 80 mL of the studied treatment in a nutritional medium of the studied food model into the stomach. The pH of these compartments is kept at 2 for the stomach and 6.8–7 for the small intestine, which has a retention time of 2 h [72,86]. The volumes present in the AC, TC and DC compartments were 250, 400 and 300 mL, respectively, which is significantly lower than those observed for previous models. In these reactors, pH is kept at 5.6 ± 0.2 in the AC, 6.3 ± 0.2 in the TC and 6.8 ± 0.2 in the DC. These values are maintained by the addition of 0.5 M NaOH and 0.5 M HCl when necessary. The temperature of the system is maintained at 37 °C and N_2_ is continuously flushed through the system to ensure anaerobic conditions. Stomach emptying is programmed to follow the equation described by Elashoff et al., 1982, with modifications depending on the consistency of the foods (liquid, semisolid or solid) [89,90,92,95,110].

For fecal inoculum preparation, the protocol described by De Boever et al., 2000, is followed, as already described for SHIME^®^ experiments. Sodium phosphate buffer (0.1 M, pH 7) is applied to dilute the fecal samples [104]. In this case, inoculation is performed with 20 mL of the fresh 20% (*w*/*v*) human fecal slurry [83,95].

For most dietary interventions, the used nutritional media have been based on the developed by Macfarlane et al., 1998, or Cueva et al., 2015, (Table 3) [83,111]. Additionally, for static fecal fermentations using this model, Colon Nutrient Medium has been used [91] (Table 3).

In DGID-CF^®^, the stomach and intestine vessels work in a discontinuous flow, while colon reactors operate under continuous conditions. The volumes for these reactors were 260 mL (stomach, ST), 410 mL (small intestine, SI), 1000 mL (AC), 1600 mL (TC) and 1200 mL (DC). Additionally, pH was kept at the specific ranges 1.7–2 (ST), 5–6 (SI), 5.5–6 (AC), 6–6.4 (TC) and 6.4–6.8 (DC). Retention times in the stomach and small intestine were 2 and 6 h, respectively. At the same time, colon compartment values were maintained as stated for the SHIME^®^ model, with an overall retention of 72 h through the colon. As for all reactors, the conditions of continuous agitation, anaerobiosis and temperature (37 °C) were controlled throughout the experiments [112].

The fecal inoculum was prepared at 20% (*w*/*v*) as described for SHIME^®^ and SIMGI^®^, with regenerated thioglycolate, which is inoculated in the colon vessels at 50 mL (AC), 80 mL (TC) and 60 mL (DC), reaching the previously stated final volume. The culture medium for this model was elaborated as in Molly et al., 1993, and Molly et al., 1994, [54,64], and 200 mL was added three times per day to the stomach vessel. When administering a complex food matrix instead of a phenolic extract, if carbohydrates and proteins are provided by the treatment, the composition of the culture medium must be modified accordingly.

An important factor in the representation of the colonic microbiota is the consideration of a single donor or multiple fecal donors. For most of the reviewed SHIME^®^ and SIMGI^®^ experiments, only one donor or one donor per experimental line is used [53,69,83,84,87,91]. However, in some studies pooled fecal samples have been used [66,70,73,76,79]. This is specially observed for TIM-2^®^ experiments, where pooled fecal samples of 3 up to 10 different donors have been used. There is still no scientific consensus on whether the optimal approach should be mixing different fecal samples or a single donor. Some authors argue that their mixing may disturb the microbial balance, inducing competition between different microorganisms for the same niche and creating a microbial community not representative of the individual pooled samples [99,113].

In addition, it may be interesting to inoculate different SHIME^®^ lines with different donors for the study of different microbiota conditions on the same treatment, as was the case with García-Villalba et al., 2017, where this allowed for the exploration of different metabolotypes [69]. Furthermore, essential information about the selected fecal donors should be indicated, as differences in microbiota have been observed due to these variables: age, gender, the previous consumption of antibiotics, or intestinal health. Moreover, most of these studies focus on the microbiota of healthy individuals. To study the role of the administration of phenolic compounds on the improvement of certain pathological conditions, the inclusion of pathology-associated microbiota could be interesting for future studies.

Overall, while conditions such as temperature or pH seem to remain constant between studies and models, other parameters such as nutritional medium composition, have shown differences, although remain similar. Specifically, reactor volumes and the volume of fecal inoculum introduced into the reactors, and therefore the relations between them, are variable.

#### 3.3.4. Dose and Administration

In the evaluation of the gastrointestinal fate of phenolic compounds, many different food matrices have been considered. Some processed foods have been evaluated in these systems as sources of these target compounds, such as fruit juices [52,73,81,103], wheat bran [93], sauces [106], red wine [83,90] and processed vegetables such as high-pressure processed onion powder [85].

However, most studies present in the literature have focused on the use of plant extracts or specific compounds isolated from these sources. In this sense, extracts from pomegranate [59], *Vitis vinifera* [52], blueberries [55,114], cranberry [82,97], *Artemisia dracunculus* (tarracon) [115], citrus fruits [100,101], green tea [100], potato [67] and red wine [116] have been used. Additionally, combinations of different extracts have also been evaluated, such as red wine/grape juice and black tea [68,71], cranberry and grape seed [117] and *Hibiscus sabdariffa* and *Aloysia citrodora* (Lemon verbena) [91]. Moreover, a current trend in the study of polyphenolic sources is the use of food industry by-products, such as olive pomace, pomegranate mesocarp [81], mango peel [98,99] or grape pomace [84,89]. Specific compounds from these sources have also been evaluated, such as soy isoflavones [76] and luteolin from oregano [70] or hesperidin 2S [75]. Additionally, specific pure phenolic compounds and combinations have also been considered [53,66]. Moreover, phenolic extracts of *Spirulina* sp. LEB-18 have also been evaluated [58].

The aforementioned are not the only administration strategies, as the co-digestion of phenolic compounds with probiotics [72,79,94,118], prebiotics [74] and other dietary compounds that may alter their bioaccessibility has also been recorded [80,88,92,112]. Moreover, the administration of microencapsulated phenolic compounds has also been considered for their dynamic gastrointestinal digestion [87]. This strategy has not yet been as thoroughly considered and thus proves to be an innovative approach in the in vitro dynamic gastrointestinal digestion of phenolic compounds.

The specific daily doses in these studies seem variable, ranging from 70 mg/day [76] to 7.5 g/day [99,106], as seen in Table 4. This may be related to the nature of the administered forms and their overall composition in phenolic compounds. Thus, doses of processed foods are usually higher than observed for commercial polyphenolic extract. In this sense, the administered dose of Mexican sauces in Cárdenas-Castro et al., 2021, was 7.5 g, while in Van Rymenant et al., 2018, 500 mg of a commercial extract was evaluated [75]. Additionally, several differences were observed for doses of phenolic compounds from *Hibiscus sabdariffa* when they were evaluated in different reactors [88,91]. Moreover, these differences were also observed for the administration of red wine, where the intake of a glass of wine was of a different volume [83,92].

Some of the approaches to the selection of a specific daily dose are through the consideration of average human polyphenolic intake, which is highly variable between different countries and the specific type of diet consumed by different populations. A European study on dietary polyphenol intake indicates the highest consumption in Denmark (with 1786 mg/day in men and 1626 mg/day in women) and the lowest in Greece (744 mg/day in men and 584 mg/day in women) [121]. In this sense, the average human consumption of polyphenols has been estimated at 1 g/day [122]. In relation to this, some studies have considered the administration of 1 g of phenolic-rich extracts [68,71]. Additionally, when specific phenolic compounds have been studied, their regular daily intake has also been considered when selecting the used doses [53].

When specific beverages or foods are administered, their daily recommended consumption may also be considered to determine the specific dose. In Wu et al., 2018, and Wu et al., 2017 [77,78], a dose of 100 mL/day of *Aronia melonacarpa* juice with 6.5 g/L of polyphenols was administered, reaching a total of 0.65 g polyphenols per day, as that was the daily recommended dose of Aronia juice from other studies [52,81,123]. In Barroso et al., 2014 [72], and Cueva et al., 2015 [83], the used dose was chosen to simulate the intake of a glass of wine.

Additionally, the previous literature on the use of certain extracts can also be considered. In Silva et al., 2022, concentrations of a commercial extract and a prebiotic were fixed according to the previous literature, where the concentration was selected considering the richness of the studied compound in the used extracts and taking into consideration the daily intake of specific phenolic compounds [91,116].

A selection of doses already recorded in in vivo trials may be helpful and has been considered [66]. In this sense, many animal trials have been carried out to investigate the relation between polyphenol intake and certain pathologies and a conversion between rat and human administration has been established [124]. Epidemiologic studies involving different intakes of phenolic-rich foods have given insight into this relation. For example, in the study by Sivaprakasapillai et al., 2009 [125], the administration of different doses of a grape seed extract, concretely 150 and 300 mg, was tested in adults with metabolic syndrome to evaluate the effect on blood pressure, observing a decrease with the administration. In van der Stelt et al., 2015, oral oleuropein supplementation with 758 mg/kg of extract reduced body weight and blood glucose in mice [126]. In Olmez et al., 2015, the administration of an ethanolic olive leaf extract at 20, 50 and 100 mg/kg/day improved the atherogenic lipid profile in rats with a high cholesterol diet [127].

However, there seems to be a lack of consistency in the reasoning behind the selection of phenolic doses. This may hinder the accurate comparison between studies, as results may not be comparable when very different doses are administered. In this sense, the inclusion of not only the justification but also the composition of the administered phenolic source may be useful. Furthermore, it must be taken into consideration that although high doses can be administered into these systems, they may not be translated into human consumption. If these doses may not be applicable to humans due to safety reasons, the demonstrated results in the previous experiments may not be observed.

Thus, in vivo polyphenol toxicity must also be considered, as these compounds have increasingly been classified as dietary hormometins [128,129]. In this sense, many in vivo animal studies have been developed to measure the potential toxicity related to oral administration, considering doses up to 5000 mg/kg/day in rats [129]. In addition, official considerations of the permitted administration of different compounds must also be considered. For example, the European Food Safety Authority (EFSA) reports an allowable daily intake value for curcumin of 0–3 mg/kg bodyweight [130].

The specific administration pattern also needs to be considered, as well as the gastrointestinal step in which the phenolic dose is included. Although treatment is usually administered through the feed, entering the stomach compartment, others consider direct introduction into the proximal colon [52,81]. In this sense, the administration of the administered dose into the stomach vessel gives us an insight into the stability of phenolic compounds during digestion and their bioaccessibility previous to the interaction of their bioaccessible fraction with the colonic microbiota [68,71,72,73,74,75,77,78,92,93]. On the other hand, direct administration into the colon region may focus on the effect of a specific extract on microbiota and may be a useful tool for understanding the effect of specific combinations of phenolic compounds and their metabolization of gut microbiota [52,81]. In addition, this strategy could also be proposed when the previous literature has established the absence of an effect of upper gastrointestinal digestion on the studied compounds’ stability. If it is not the case, the degradation of these compounds throughout the gastrointestinal tract could not be considered, which may result in a lack of information regarding the real metabolization, modulation and bioactivity of those extracts.

Another factor in the selection of a specific dose is the effect of the digestive conditions on the final colonic concentration. Differences in vessel volumes between different models and the published literature, as well as the inoculated fecal slurries, influence microbiota abundance and the final concentration of the final dose that gets into this area [72,83,88,91,92]. Thus, these differences may present changes in observed results and must be carefully studied and considered.

As for the administration pattern into the system, in some cases, the culture medium was directly supplemented with the selected dose [73]. However, two main strategies have been observed. In the first one, the complete dose is administered only once a day [79,98]. This strategy is also observed in acute studies [68]. On the other hand, the distribution of the dose into three daily administrations is also possible [68]. In this regard, one of the differences between these two approaches is the achieved concentration of phenolic compounds throughout different moments in the digestive process. A single dose may allow for a higher concentration during a specific time, while a more frequent administration with a lower quantity each time would ensure a stable concentration during the process.

Finally, multiple doses and administration strategies have been considered throughout the literature. This variability could hinder comparison between studies. Thus, selection of the specific dose should consider different factors such as the daily recommended intake of specific foods or compounds, mean consumption, toxicity or the previous literature.

### 3.4. Sample Treatment

Samples can be taken throughout the whole digestive process. As such, the frequency and the optimized treatment of the samples before analyses must be considered. Overall, samples are collected at regular intervals, depending on the nature of the study. For short-term experiments, samples are taken every 24 h, or times could be further reduced into 1–4 h periods [66,73,80,88,89,91,92,99,101,106]. This reduction can be higher in shorter experiments and samples could be taken every 15–30 min [87]. On the other hand, in long-term experiments, samples are usually taken from 1 to 3 times per week from the upper gastrointestinal tract and each colon section [53,74,77,78,81]. In most studies, samples are taken once a week for microbial community analysis by selective enumeration, which is performed at the time of sampling [68,74,83,91]. The mentioned considerations are also applicable to acute and chronic experiments [83,84].

The volumes obtained each time can depend on the intended posterior assays and the studied digestive step. Thus, variability in this parameter is also observed, with volumes ranging from 5 mL [73] to 20 mL [81] or 40 mL [55].

Additionally, it is necessary to contemplate the sampling at different phases. To establish a connection between administration and observed effects, baseline samples throughout the control period or from a control line in the system must be taken. The consideration of the washout period may allow for insight into the duration of the residual effect of the treatment [76].

As analysis is rarely performed at the time of sampling, samples are subsequently stored and refrigerated once collected at −20⁰C or −80⁰C [53,66,68,69,71,72,77,78,79,80,81,87]. Moreover, the freeze-drying of samples has also been considered [93]. In some cases, samples were centrifuged previously to their storage [70,78,81,87,88,99]. In this sense, supernatants and pellets were used for different purposes: the former were intended for phenolic analysis, Short Chain Fatty Acid (SCFAs) and ammonium determinations while the later for microbial genetic analyses [83,84,89,91,92,95]. In some cases, pellets are directly stored for their later DNA extraction [78] or genetic material extracted can even be conserved at that temperature for later analysis [52,76,81]. This may also be used to obtain bioaccessible and non-bioaccessible fractions. However, in TIM^®^ experiments, this may not be necessary as these fractions are directly obtained from the equipment.

It is also important to consider the treatment of these samples before their analysis, specifically for the identification and quantification of phenolic compounds. When studying the administration of bioactive compounds in such small dosages in high-volume reactors and vessels, it is important to take into consideration their diluting effect. This fact may limit the information we obtain from samples taken throughout the process of phenolic composition.

Additionally, for the phenolic characterization of many complex matrices, some studies have included the extraction of phenolic compounds from the digested samples. This has been mainly achieved using liquid–liquid extractions with organic solvents such as diethyl ether [73], ethyl acetate [75,100] or methanol [55]. This extraction may be followed by an evaporation and congelation to remove all the remaining water and then re-extraction [100]. Furthermore, the extracted mixture may be centrifuged (4000× *g*, 20 min, 4 °C) and the supernatant evaporated for their later resuspension in the analytical solvent [55]. Due to the diluting effect previously mentioned, the sample treatment could include a preconcentration step in order to achieve the limits of detection and quantifications of the selected analytical technique, further discussed in Section 3.6.

Additionally, the defattening of the residue and efflux samples from TIM-2^®^ was performed using 2 mL hexane and later partitioned 3 times with 2 mL of ethyl acetate, which was later evaporated [115].

As can be observed, a variety of protocols can be followed for the treatment of samples once obtained, which may hinder comparability between studies. Hence, the development of a consensus in this area is needed.

### 3.5. Microbiological Analyses

Different microbiological analyses have been applied to study microbiological diversity in these dynamic systems and to record the differences caused by the administration of different phenolic combinations. Two main approaches can be followed: an indirect study through microbial metabolism or an identification and quantification of the present microbial communities.

**Microbial metabolism**. One of the main studied products of the microbial metabolism are SCFA, although Branched Chain Fatty Acids (BCFAs) have also been considered [72,79]. For their extraction from digested samples, diethyl ether and the addition of 2-methyl hexanoic acid as internal standard has been used [68,69,72,74,75,82], based on the protocols proposed by Possemiers et al., 2004, and De Weirdt et al., 2010 [102,131]. In addition, extraction with diethyl ether can be performed after the addition of H_2_SO_4_, as described in Greenberg et al., 1992 [52,81,132]. Additionally, extraction using sodium chloride, chrotonic acid, isobutanol and H_2_SO_4_ has also been performed [79]. The most used analytical platform for the analysis of these compounds is gas chromatography with a flame ionization detector (GC-FID) or coupled to mass spectrometry (GC-MS). Indeed, in most SIMGI^®^ experiments, SCFAs were extracted using H_2_SO_4_ and 2-methylvaleric acid as internal standard and analyzed by solid phase micro-extraction (SPME) and GC-MS as described in Cueva et al., 2015 [83]. In addition, proton Nuclear Magnetic Resonance (H^1^ NMR) profiling has also been used [71].

Ammonium is another indicator of microbial activity, and its determination throughout studies seems to be similar. Ammonium levels were determined by the release of this species as ammonia by the addition of MgO, its distillation into boric acid solution and subsequent back-titration. Additionally, this analysis can be made using an anion measurer attached to an ion-selective electrode [53,79,83,94], a commercial kit for ammonium determination [91,92,95] or enzymatic quantification using the Berthelot reaction [98].

Additionally, lactic acid levels have also been established as indicators of microbial metabolism, although they seem to be less used in general. Its analysis is based on the use of commercially available enzymatic assay kits [72,73,74,75,76], although in the past ion chromatography was also used for the detection of lactic acid levels in order to evaluate microbial metabolism [133,134,135].

**Microbial communities**. The use of selective plating techniques may be a broad approach in the determination of the microbiota, as it gives us an estimate of the number of culturable bacteria [53,68,72,73,83,91,92,94,95,103]. For this purpose, decimal dilutions of samples in a physiological solution (0.9%) have been used for plating on different culture media, as can be observed in Table 5.

However, the use of this approach may present some limitations, as it only gives information on culturable strains. The development of new techniques focused on the molecular fingerprinting of these microbial communities has provided additional information through the inclusion of non-culturable strains and pose as interesting alternatives [102].

For this purpose, the extraction of microbial DNA is needed and can be achieved using a lysis buffer and glass beads [52,68,72,81,117] and the later use of phenol-chloroform [52,73,74,81] or cetyltrimethylammounium bromide (CTAB) buffer and phenol-chloroform-isoamyl alcohol [68,72,117]. In some cases, EtOH/NaOAc can be used for precipitation [52,81]. Alternatively, extraction can be achieved using commercial kits, such as TIANamp Stool DNA Kit [76], QIAamp Fast DNA Stool Mini Kit [79,83,84,91,103], Powerfecal DNA isolation kit [69], AGOWA mag Mini Kit [113] or DNeasy PowerSoil Kit [96,112].

Another methodology for the identification and quantification of bacterial communities is the quantitative polymerase chain reaction (qPCR) or real time PCR. This technique has been used for the quantification of different bacteria groups using different bacterial DNA such as total bacteria (DNA from *Escherichia coli* DH5α, *E. coli* ATCC 25922, *E. coli* CECT 515), *Lactobacillus* (*L. plantarum* IFPL935, *L. plantarum* CECT 748), Bifidobacterium (*Bifidobacterium breve* 29 M2, *Bifidobacteirum longum* DSM 20088), Firmicutes (*Clostridium leptum* DSM753), Bacteroidetes (*Bacteroides ovatus* DSM 1896), or Bacteroides (*Bacteroides fragilis* DSM2151) [69,72,83,117]. Additionally, quantification can also be performed using standards derived from targeted cloned genes, using cloning vector systems kits [72].

Quantification has targeted total bacteria [68,69,72,83,84,95], *Lactobacillus* [72,74,84,95], Bifidobacterium [68,69,74,84,95], Bacteroides [68,69,72,83,84,95], Enterobacteriaceae [84,95], Enterococcaceae [84,95], *Clostridium coccoides–Eubacterium rectale* Cluster XIVa [75,84,95], *Ruminococcus* Cluster IV, *Clostridium leptum* subgroup specific cluster IV [72], Firmicutes, *Bacteroidetes*, Akkermansia [69,74], Gordonibacter [69], *Faecalibacterium prausnitzii* [69,74,95], *Blautia coccoides* [68].

The analysis of different hypervariable regions of the microbial 16S rRNA gene has also been performed for this purpose: V3-V4 [68,69,78,90,91,95,96,98,101,109,112]. For this purpose, samples have been submitted to 2 × 500 bp [61,63] or 2 × 300 bp [91,96,112] paired-end sequencing using an Illumina^®^ MiSeq instrument or performed by BaseClear [69] or Cogenics/Beckman genomics [68].

Polymerase chain reaction denaturing gradient gel electrophoresis (PCR-DGGE) has been widely used in SHIME^®^ experiments and mainly for the identification/quantification of total bacteria and specific bacterial groups. The primers used for total bacteria were 338F-GC and 518R [52,68,81], although 968FGC and 1401R have also been used [79,103]. Other bacterial groups targeted were lactic acid bacteria (SGLAB0159F/SGLAB0667R), Bacteroides/Prevotella (FDI/rP2), Bifidobacteria (Bif164-F/Bif662-R), *L. plantarum* (Lab-159 F/Uni-515-GC-R) [52,72,73]. For DGGE, an 8% polyacrylamide gel with a 45–65% denaturing gradient in a 1× TAE buffer (20 mM TRIS, 10 mM acetate, 0.5 mM EDTA pH 7.4) has been used for total bacteria [52,68,69,103,105]. Other denaturing gradients have been applied for other bacterial groups, such as 50–65% of Bifidobacteria [82] or 30–50 % of 7 M urea and 40% formamide for *L. plantarum* [101].

### 3.6. Phenolic Compound Identification and Bioaccessibility

As these studies consider the dynamic digestion of phenolic compounds, different methodologies can be applied for their identification and quantification. As a broader approach, the determination of the total phenolic content (TPC) through the Folin–Ciocalteu method [73,92,95] has been used. Nevertheless, High Resolution Liquid Chromatography (HPLC) coupled to different detection techniques has been the main strategy: mainly diode-array detector (DAD) [93], or mass spectrometry (MS) with their multiple analyzers via an electrospray interface (ESI), concretely tandem quadrupole detector (TQD-MS or MS/MS) [72,83,84,89,90,92,95,117,136] and quadrupole-time of flight analyzer (QTOF-MS) [88,91,99,106], whereas the coupling of both detection techniques was also applied [52,81]. In addition, although they are not commonly used in these studies, other techniques could be applied for this purpose, such as gas chromatography coupled to mass spectrometry (GC-MS) for the identification and quantification of phenolic acids [71,100] and sterols [98,109,112], and matrix-assisted laser desorption/ionization time of flight mass spectrometry (MALDI-ToF-MS) [137,138].

In this sense, among the different modes of HPLC, reverse phase has been the most used separation, through the use of non-polar C18 columns with different characteristics: from the longer and higher particle size columns of the Phenomenex Luna C18 (4.6 × 150 mm, 5 μm; Phenomenex, Torrance, CA) [71,85,91], Ascentis Express C18 (3.3 × 150 mm, 2.7 μm; Sigma-Aldrich Quimica, Madrid) [88,99,106], Poroshell RP-18 (3 × 150 mm, 2.7 μm; Agilent Technologies, Santa Clara, CA) [81] and Alltima C18 (7 × 53 mm, 5 μm; Alltech) [100] to the shorter and smaller particle size of the Waters Acquity UPLC BEH shield RP18 (3 × 150 mm, 1.7 μm; Waters, Milford, MA) [75] or Waters BEH C18 (2.1 × 100 mm, 1.7 µm; Waters, UK) [70,72,83,84,89,92,95,116,117,136]. Regarding the mobile phases, the most common solvents were mixtures of water with acid modifiers and organic solvents such as methanol or acetonitrile in different proportions along the analysis following a multistep gradient.

As for the identified compounds, this has varied depending on the specific focus of the study as well as the applied phenolic source. This has included: flavonoids and their metabolites, such as anthocyanins and flavan-3-ols [84,91,92,116,136], phenylpropanoids [91], and phenolic derivates, among others [72,85,95,116].

The bioaccessibility of phenolic compounds through the digestive tract is an important factor of study. Thus, the evolution of phenolic compounds can be expressed through their concentration at different times and at different compartments along the digestive process [52,72,73,84,88,91,99]. Moreover, this parameter can also be expressed as percentage of total polyphenol recovery after gastrointestinal simulation in relation to the total content present in the administered dose [90,93]. Different formulas have been applied for this purpose throughout different studies. For example, in Tamargo et al., 2022 [95], phenol recovery (%) = [(total phenolic content in the 24 h cranberry–effluent × V_effluent_/ total phenolic content in the 24 h cranberry–feed × V_feed_)] × 100].

The recovery of phenolic compounds has also been calculated for transport experiments using the Caco-2 cell model. In this sense, apical and basal recovery (%) have been stated as (concentration after transport)/(initial concentration of apical side at 0 h) × 100 [77].

In summary, similitudes in this area are observed. Nevertheless, the inclusion of studies concerning the selection of an analytical methodology focused on the study of phenolic compounds may be interesting for further standardization.

### 3.7. Antioxidant Activity of Samples and Digestates

To evaluate the antioxidant capacity of the studied samples, the antioxidant compounds studied were extracted when needed [60,85,103]. That was the case for Duque et al., 2016, where antioxidant compounds present in orange juice were extracted with a methanol:water solution (80:20 *v*/*v*). Additionally, samples from the digestive simulation process were pre-treated through centrifugation and diluted prior to the antioxidant assay [103].

The most used antioxidant method has been ABTS. As for methodology, different volumes of sample (2–100 µL) and ABTS solution (0.29–3 mL) have been used [56,60,66,79,85,103]. To study the antioxidant activity of digestates, DPPH, FRAP and enzyme assays have also been applied [57,60,85].

These methods have been proved as adequate for the evaluation of the antioxidant capacity of compounds throughout the digestive simulation process. The results showed a decrease in antioxidant activity in the gastric digestates with a later increase during intestinal digestion [85]. Meanwhile, in the colon, the administration of phenolic compounds showed a significant increase in antioxidant activity in comparison to control conditions [79,103]. Additionally, taking samples at different digestion times after the introduction of the phenolic compounds allows us to study an antioxidant profile and relate it to the presence and metabolization of phenolic compounds. In Ekbatan et al., 2016 [66], although an increase in activity was observed in the AC at the beginning of digestion, this was only observed in TC and DC after 16 h of digestion, which represents the importance of the metabolization of parent phenolic compounds by the colonic microbiota in the overall antioxidant activity of phenolic compounds. In Duque et al., 2016 [103], the presented antioxidant activity was similar to that present in the evaluated juice. These models could be interesting for the evaluation of the antioxidant activity of the administration of these compounds as alterations in these values due to the food matrix effect can be studied [56].

In addition to assessing the release of polyphenols and their impact on microbial diversity, the study by Attri et al., 2018 [73], also analyzed the total antioxidant activity (TAC) of polyphenol-rich sea buckthorn berry juice. The total polyphenol content (TPC) of the different samples collected at different time intervals from different reactors was determined by the Folin–Ciocalteu method. The TAC of the different digested fractions was carried out using the ABTS method proposed by Pellegrini et al. [139]. After the gastric and small intestine phase, TPC values were found to be 248.56 mg GAE/l and 325.86 mg GAE/l, respectively. For TAC, they found values of 21.47 TEAC mM/l and 26.76 TEAC mM/l after the gastric and small intestine phases. Finally, the highest concentrations of TPC (431.45 ± 21.1 mg GAE/l) and TAC (39.73 ± 1.9 TEAC mM/l) were observed in the descending colon. In this sense, similar results were found by Duque et al., 2016 [103] reporting an increase in TAC and TPC in all the three regions of the colon.

The antioxidant capacity of soy isoflavones using the SHIME system was recently analyzed by Chen et al., 2022 [76]. For this purpose, three different assays were carried out: DPPH, ABTS (both expressed as % inhibition rate) and FRAP (expressed as vitamin C equivalents (mg VcE/g). The results obtained by the three methods were similar. There were no significant changes in antioxidant capacity in the oral cavity and small intestine. During the digestion phase in the stomach, the inhibition rates of DPPH and ABTS and FRAP values decreased from 64.11 ± 1.83%, 60.36 ± 3.18% and 15.60 ± 0.94 mg VcE/g to 53.34 ± 1.02%, 51.27 ± 1.28% and 12.30 ± 1.03 mg VcE/g, respectively. Finally, a significant reduction in the antioxidant capacity of isoflavones was observed in the colonic phase.

## 4. Conclusions

In recent years, several tools for the evaluation of the digestive process on the phenolic compound profile, metabolization and modulation of the colonic microbiota have been developed. This review has recorded all the differences found between some of the most used dynamic gastrointestinal models for the study of phenolic compounds. Although a standardized and harmonized protocol for static in vitro digestion has been described, its translation into dynamic in vitro digestion processes is quite complex due to the described differences between models. However, a series of guidelines regarding factors to be considered in the development of one of these studies could be proposed to enhance the comparability between studies and models.

Firstly, it could be useful to stablish a minimum and maximum treatment period to observe the effects on the colonic microbiota. Digestive conditions seem to be similar between the reviewed studies, but volumes per digestive stage were variable, although proportions were maintained. In this sense, an evaluation of the differences in fecal inoculum volume introduced in relation to those different reactor volumes might be interesting, as the standardization of this administration throughout models could allow for a better understanding of the microbiota.

A standardization of the administered dosage might also be interesting for ensuring comparability purposes and the observation of realistic human administrations. Factors such as the daily recommended intake of foods or compounds, mean consumption, toxicity and information from clinical trials should be considered in this area. When phenolic extracts are used, the dosage at which they are included in functional foods or formulations could be considered, as the observed results should be representative of an applicable dose. Moreover, establishing doses that guarantee the delivery of a specific concentration in the colon may allow for a better comparability between studies.

As has been described in detail, sample treatments, microbiological analyses and analytical evaluations are similar between studies. However, it could be useful to develop a recommended protocol for studying phenolic compounds. Specifically, studies on the selection of sample treatment conditions, which are a critical step, and analytical methodologies focused on the study of phenolic compounds could be developed. Regarding antioxidant analysis, the above-mentioned studies suggest the need for further research focused on the evaluation of the effect of dynamic in vitro digestion on the antioxidant capacity of compounds present in foods at the gut microbiota level, as there are few studies analyzing this aspect.

Additionally, further research could be performed on approaches such as the use of pathology-related microbiota, encapsulated formulations and co-administration with probiotic strains.

## Figures and Tables

**Table 1:** Nutritional media composition of the administered feed to the SHIME model for the study of phenolic compounds, present throughout all digestive compartments.

Feed Components	Concentration (g/L)	References
Arabinogalactan	1.0	[71,72,73,75,76,81,102,103]
	1.2	[39]
Pectin	2.0	[71,72,73,75,76,81,102,103]
Xylan	1.0	[71,72,73,75,76,81,102,103]
	0.5	[70]
Starch	4.0	[70,75,76,80,81]
	3.0	[71,72,73,77,78,79,80,81,82,83,84,85,86,87,88,89,90,91,92,93,94,95,96,97,98,99,100,101,102]
Glucose	0.4	[70,71,72,73,75,76,81,102,103]
Yeast Extract	3.0	[70,71,72,73,75,76,81,102,103]
Peptone	1.0	[70,73,76,77,81,102,103]
	3.0	[69,74,75,80]
Mucin	4.0	[71,72,73,76,81,102,103]
	1.0	[69,74,75,80]
	0.3	[70]
L-Cysteine Hydrochloride	0.5	[70,71,72,73,75,76,81,102,103]

**Table 2:** Nutritional media composition of the SIEM in different studies.

Components of the Feed	g/L			
Pectine	12 ^a^	9.4 ^b^	9 ^c^	4.7 ^d^
Xylan	12 ^a^	9.4 ^b^	9 ^c^	4.7 ^d^
Arabinogalactan	12 ^a^	9.4 ^b^	9 ^c^	4.7 ^d^
Amylopectin	12 ^a^	9.4 ^b^	9 ^c^	4.7 ^d^
Starch	100 ^a^	78.4 ^b^	74.6 ^c^	39.2 ^d^
Tween 80	270 ^a^	34.0 ^b^	31.5 ^c^	17 ^d^
Bactopepton	375 ^a^	47.0 ^b^	43.7 ^c^	23.5 ^d^
Casein	375 ^a^	47.0 ^b^	43.7 ^c^	23.5 ^d^
Ox-bile	6.25 ^a^	0.8 ^b^	0.7 ^c^	0.4 ^d^
MgSO_4_	50 ^a^	0.5 ^b^	0.7 ^c^	-
L-Cysteine	20 ^a^	0.4 ^b^	0.3 ^c^	-
Vitamin mixture	-	1 mL ^b^	1.5 mL ^c^	-
K_2_HPO_4_·3H_2_O	4.7 ^a^	2.5 ^b^	4.7 ^c^	-
NaCl	8.4 ^a^	4.5 ^b^	8.4 ^c^	-
CaCl_2_·2H_2_O	0.8 ^a^	0.45 ^b^	0.8 ^c^	-
FeSO_4_·7H_2_O	0.009 ^a^	0.005 ^b^	0.009 ^c^	-
Haemin	0.02 ^a^	-	0.02 ^c^	-

References: ^a^ [101,106,109]; ^b^ [99]; ^c^ [88,98,99]; ^d^ [93].

**Table 3:** Nutritional media composition of the administered feed to the SIMGI model, including Colon Nutrient Medium used for colon-only experiments.

Feed Components	Concentration (g/L)	References	Colon Nutrient Medium (CNM)	Concentration (g/L)	References
Arabinogalactan	1	[83,84,92]	Yeast Extract	2	[91]
Pectin (from apple)	2		Peptone	2	
Xylan	1		L-cysteine	0.5	
Potato Starch	3		NaCl	0.1	
Glucose	0.4		K_2_HPO_4_	0.04	
Yeast Extract	3		KH_2_PO_4_	0.04	
Peptone	1		MgSO_4_·7H_2_O	0.01	
Mucin	4		CaCl_2_·6H_2_O	0.01	
L-cysteine	0.5		NaHCO_3_	2	
			Tween 80	2 mL/L	
			Hemin	0.05	
			Vitamin K	10 µL/L	
			Bile salts	0.5	

**Table 4:** Doses, selection, fecal donors and references for reviewed articles.

	Reference	Dose	Selection	Fecal donors	Protocol source
Single SHIME	[73]	10% of juice/day	-	Pool (four donors)	-
	[74]	1597 mg/day	A commercial extract, which indicated composition	one donor	[102]
	[76]	70 mg isoflavones/day	-	Pool (three donors)	-
	[77]	100 mL/day juice with 6.5 g/L polyphenols	Daily recommended dose of Aronia Juice (Kardum, 2017)	one donor	[102]
	[78]	100 mL/day juice with 6.5 g/L polyphenols	Daily recommended dose of Aronia Juice (Kardum, 2017)	one donor	[102]
	[79]	200 mL formulation/day	-	Pool (three donors)	[102]
	[80]	2.18 µM of polyphenols	-	-	-
	[94]	2.5% (*w*/*v*) of by-product	As the minimum level established by the Brazilian Health Regulatory Agency for food to be considered as a source of fiber.	Pool (three donors)	-
	[119]	50 g cooked beans and 50 g corn-tortilla	-	Pool (three donors)	[102]
	[118]	500 mg extract/L	-	one donor	[102]
	[68]	1 g extract	Content present in red wine and grape juice extract and tea extract from Lipton’s black tea	one donor	[102]
TWIN- SHIME	[69]	1.8 g/day	-	one donor per metabolotype	[102]
	[70]	1.5 g oregano, 9.7 mg luteolin/100 g	-	Pool (three donors)	[102]
	[71]	1 g extract	Polyphenol doses selected to simulate physiological conditions since total intake for humans has been estimated at 1 g/day	one donor	[102]
	[72]	200 mg extract/day; 200 mg extract/day + 1010 CFU Lactobacillus	It corresponds to daily polyphenol intake of 250 mL of red wine	one donor	[104]
	[75]	500 mg of Cordiart (450 mg hesperidin)	-	one donor	-
	[87]	5 mg of curcuminoids, 500 mg of microencapsulated, 250 mg of turmeric and 25 mg of Meriva^®^.	-	one donor	[70]
	[52]	1 y 2 g extract/L	Taking into consideration previous human studies with stilbenes	one donor	[102]
Triple- SHIME	[81]	2–4 g/L of extract	-	one donor	[102]
	[53]	200 mg of (+)-Catechin	The dose represents a regular intake (+)-catechin in humans	Pool (12 donors)	[104]
	[82]	7.4 g/L extract	-	Pool (five donors)	-
	[117]	500 mg/L	-	-	[74]
Other	[83]	225 mL, corresponding to 405 mg of polyephnols	To simulate the intake of a glass of wine	1 donor per experiment (two donors)	[104]
SIMGI	[84]	700 mg (acute and chronic)	-	one donor per experiment (two donors)	[104]
	[91]	90 mg and 270 mg of extract	According to Sánchez-Patán, 2012; Cueva et al., 2013	one donor per experiment (two donors)	-
	[89]	1 g extract (47.96 mg GAE/g phenolics)	-	one donor	[92]
	[92]	1 g of extract	-	one donor	[84]
	[95]	240 mL wine and 80 mL of prepared food models.	Considering a daily dose of 240 mL of wine (Cueva, 2015, Muñoz Gonzalez, 2013)	one donor	[84]
	[90]	80 mL wine (200 mg of gallic acid equivalents)	-	one donor	[92]
	[55]	0.5 of extract with 100 g of meal matrix	-	-	-
TIM-1	[114]	134.5 mg, 403.5 mg and 2 g of extract	-	-	-
	[115]	0.5 g of extract	-	-	-
	[55]	20 mg rosmarinic acid	-	-	-
	[106]	7.5 g/day, 2.5 mL/h two Mexican sauces	-	Pool (seven donors)	[78]
TIM-2	[100]	600 mg extract	-	Pool (10 donors)	-
	[73]	250 y 350 mg extract/day	-	Pool (seven donors)	[113]
	[98]	7.5 g/day, 2.5 mL/h pre-digested mango peel	-	Pool (three donors)	[105]
	[120]	7.5 g/day, 2.5 mL/h pre-digested mango peel	-	Pool (three donors)	[105]
	[88]	-	-	Pool (three donors)	[105]
	[99]	7.5 g/day, 2.5 mL/h pre-digested mango peel	-	Pool (three donors)	[105]

**Table 5:** Culture media used for analysis of microbial communities.

Target Group	Selective Culture Media	Incubation Time	Incubation Conditions	Reference
Total aerobes	Brain heart infusion (BHI) Agar	24	Aerobic	[68,73]
	Standard Methods agar	48	Aerobic	[103]
	Trypticase Soy Agar (TSA)	48	Aerobic	[83,84,91,92,95]
Total anaerobes	Brain heart infusion (BHI) Agar	72	Anaerobic	[68,72]
	Standard Methods agar	48	Anaerobic	[103]
	Wilkins-Chalgren agar	48	Anaerobic	[83,84,91,92,95]
Lactic acid bacteria	De Man, Rogosa and Sharpe (MRS) Agar	48	Aerobic	[72,73]
		48	Anaerobic	[83,84,91,92,95,103]
Lactobacilli	LAMVAB Agar	48	Aerobic	[72]
		72	Microaerophilic incubation	[68]
		48	Anaerobic	[91,92,95]
*Bifidobacterium* spp.	Bifidobacteirum agar modified by Beerens (Difco., BD, USA)	48	Anaerobic	[91]
	BIM-25 agar	72	Anaerobic	[103]
Total Coliforms	MacConkey agar	24	Aerobic	[68,72,73]
Staphylococci	Mannitol Salt agar broth	48	Aerobic	[68,84,95]
*Clostridium* spp.	Reinforced Clostridial agar	48	Anaerobic	[103]
	Tryptose Sulfite Cycloserine (TSC)	48	Anaerobic	[91,92,95]
Enterobacteria	MacConkey agar	48	Anaerobic	[83,84,91,92,95]
*Enterococcus* spp.	KF Streptococcus agar	48	Aerobic	[103]
	Enterococcus agar	48	Anaerobic	[84,91,92,95]
	Enterococcus agar	48	Aerobic	[68]
	Bartley and slanz agar	48	Aerobic	[73]

## Data Availability

Not applicable.
